# Innovative Analyses Support a Role for DNA Damage and an Aberrant Cell Cycle in Myelodysplastic Syndrome Pathogenesis

**DOI:** 10.1155/2011/950934

**Published:** 2011-06-07

**Authors:** David R. Head, James W. Jacobberger, Claudio Mosse, Madan Jagasia, William Dupont, Stacey Goodman, Leanne Flye, Andrew Shinar, Sara McClintock-Treep, Greg Stelzer, Robert Briggs, Keith Shults

**Affiliations:** ^1^Vanderbilt University Medical Center, Nashville, TN 37232-5310, USA; ^2^Case Comprehensive Cancer Center, Cleveland, OH 44106-5065, USA; ^3^Tennessee Valley Healthcare System, U.S. Department of Veterans Affairs, Nashville, TN 37212, USA; ^4^Esoterix Center for Innovation, Brentwood, TN, USA

## Abstract

We used flow cytometry to analyze the cell cycle, DNA damage, and apoptosis in hematopoietic subsets in MDS marrow. Subsets were assigned using CD45, side scatter, CD34, and CD71. Cell cycle fractions were analyzed using DRAQ 5 (DNA content) and MPM-2 (mitoses). DNA damage was assessed using p-H2A.X, and apoptosis using Annexin V. Compared to controls, MDS patients demonstrated no increased mitoses in erythroid, myeloid, or CD34+ cells. Myeloid progenitors demonstrated increased G2 cells, which with no increased mitoses suggested delayed passage through G2. Myeloid progenitors demonstrated increased p-H2A.X, consistent with DNA damage causing this delay. Annexin V reactivity was equivalent in MDS and controls. Results for each parameter varied among hematopoietic compartments, demonstrating the need to analyze compartments separately. Our results suggest that peripheral cytopenias in MDS are due to delayed cell cycle passage of marrow progenitors and that this delayed passage and leukemic progression derive from excessive DNA damage.

## 1. Introduction 

Myelodysplastic syndrome (MDS) is characterized by life-threatening peripheral blood cytopenias and a propensity to progress to acute myeloid leukemia (AML). Pathogenetic explanations for both characteristics remain elusive. MDS is a serious health problem, especially in the expanding elderly population, where incidence approaches 80 cases per 100,000 population per year [1–5]. There is no effective curative strategy for MDS in elderly patients, and in younger patients curative treatment consists of allogeneic stem cell transplantation, which is expensive with associated morbidity and mortality [6–8]. A current pathogenetic model of MDS is hyperproliferation of marrow progenitors but poor production of circulating cells due to excessive *in vivo* apoptosis; however, this model is not supported by the absence of hyperuricemia as a defining characteristic of the disease and fails to explain the propensity of MDS to progress to AML. An alternative model is that MDS is inherently a mutator phenotype characterized by increased DNA damage, that causing impaired cell cycling, failure of production of peripheral blood cells, and leukemic transformation. Improved treatment strategies for MDS require clarification of its pathogenesis. To investigate these issues we used multiparametric flow cytometry to analyze the cell cycle, including mitotic events, DNA damage, and apoptosis in individual hematopoietic precursor compartments in marrow samples from patients with MDS. 

## 2. Methods

### 2.1. Patients

MDS patients receiving no current treatment were studied. Diagnosis of MDS was based on review by three observers of peripheral blood and marrow morphology, clinical history, laboratory data, and cytogenetics. Diagnoses and classification were based on published recommendations and were blinded to study results [9–11]. Patients undergoing elective orthopedic procedures with no marrow-based disease were used as controls.

### 2.2. Samples

**Figure 1:**
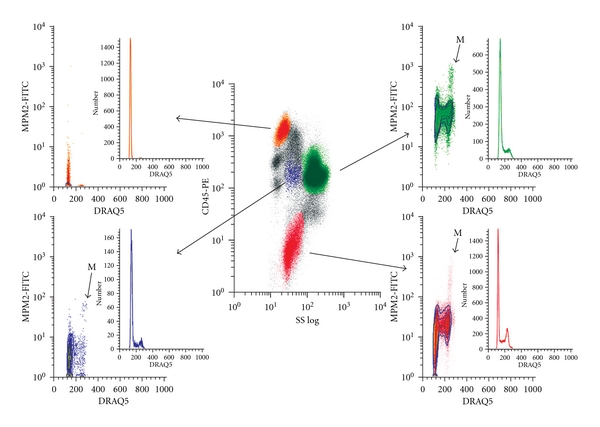
Multiparametric flow cytometry analysis of representative control bone marrow. Multiparametric flow cytometry analysis of control bone marrow displaying major subsets of cells based on SSC, CD45 density, CD34 (not shown), and CD71 (not shown) (center panel). DNA content (DRAQ5) analysis is plotted versus number of cells or versus MPM2 signal intensity for each of the gated populations (large arrows). The lymphocyte population (orange) (high CD45, low SSC) (upper left) contains predominantly cells with G1 cell cycle phase DNA content and no mitoses. The stem cell (blue) (intermediate CD45 and SSC, CD34+) (lower left), myeloid (green) (intermediate CD45, high SSC) (upper right), and nucleated erythroid (red) (intermediate SSC, low CD45, CD71+, DRAQ V+) (lower right) populations contain cells with G0/G1, S, and G2/M DNA content, and cells that mark for mitosis (M) (elevated MPM2 signal in cells with G2/M DNA content).

MDS samples were collected from marrow aspirates performed for routine clinical purposes. Controls were obtained at the time of surgical insertion of orthopedic implants. After collection, samples were diluted 1 : 1 with RPMI. Samples were analyzed at 2–5 hours after procurement (mean 3 hours). Controls samples used for Figures 1, 3, and 4were obtained from patients with uninvolved marrow undergoing marrow staging for a solid tumor. 

### 2.3. Flow Cytometry

Following Ficoll-Hypaque isolation of nucleated marrow cells, samples were assessed for CD45, CD34, and CD71 surface antigen density, log side scatter, DNA content (DRAQ5), mitotic index (reactivity with MPM-2, a monoclonal antibody against a phosphorylated epitope found on several proteins, including DNA topoisomerase II, cdc25, and Ki-67, phosphorylation being a marker of passage through mitosis), phosphorylation of histone H2A.X at serine 139 (p-H2A.X reactivity, a marker of DNA double-strand breaks), and Annexin V binding (marker of programmed cell death), using an FC500 (Beckman-Coulter, Fullerton, Calif) flow cytometer [12–14]. Major marrow subpopulations (myeloid, erythroid, CD34+, lymphoid) were distinguished using CD45, side scatter, CD34, and CD71. Antibodies to CD45, CD34, and CD71 were purchased from Beckman-Coulter; MPM-2 from Dako (Carpinteria, Calif), p-H2A.X from Cell Signaling Technologies (Danvers, Mass), Annexin V from Invitrogen (Carlsbad, Calif), and DRAQ5 from Apotech (San Diego, Calif).

### 2.4. Identification of Marrow Populations

DRAQ5 uptake was used to select nucleated cells. Hematopoietic CD34+, lymphoid, myeloid, and erythroid cell populations were then discriminated by CD45/side scatter [15] coupled with secondary gating on CD34 and CD71 surface antigen expression. Criteria for subpopulation identification were nucleated erythroid lineage (CD71+/CD45-neg/low side scatter), myeloid lineage (moderate CD45+/high side scatter), stem cells (CD34+/moderate CD45+/low side scatter), and lymphoid lineage (CD34-neg/CD71-neg/CD45++/low side scatter) [15].

### 2.5. Cell Cycle Analysis

DRAQ5 staining was used for DNA content analysis of cell cycle fractions (G0/G1, S, and G2/M), allowing cell cycle fraction analysis in each major marrow subpopulation using CD45, side scatter, CD34, and CD71 as described [13, 15–18]. DRAQ5 does not require fixation and membrane permeabilization for DNA assessment. Cell cycle fraction expression of p-H2A.X and MPM-2 in each major marrow subpopulation was achieved using cell surface marker staining (CD45, CD34, CD71), followed by fixation/permeabilization of the cells in PermiFlow (InVirion, Oak Brook, Ill), followed by intracellular antigen (p-H2A.X and MPM-2) and DRAQ5 staining [19]. Cell cycle fraction expression of Annexin V in each major marrow subpopulation was achieved by combining Annexin V reactivity with CD45, side scatter, CD71, CD34, and DNA content (DRAQ5) in an unfixed state in the presence of HBSS (Mediatech, Inc. Herndon, Va). 

### 2.6. Data Acquisition and Analysis

Each cytometry file contained 250,000 total events. Primary gating and fractional analysis was performed using WinList 5.0 software (Verity Software Topsham, Me) with DDE links of each cell population to ModFit LT 3.0 (Verity Software) for cell cycle modeling. The analysis results of each population were ported into Microsoft Excel (Redmond, Wash) for additional analysis. For p-H2A.X reactivity, positive controls were marrows challenged with nitrogen mustard to induce DNA damage. For Annexin V reactivity, negative controls were based on measurements in Ca^++^, Mg^++^ free media, as these ions are necessary for Annexin V reactivity. Analyses were blinded to clinical results.

### 2.7. Institutional Review Board: Review and Approval

Studies were reviewed and approved by the Vanderbilt University Institutional Review Board.

### 2.8. Statistical Analysis

For each cell type the proportions of cells from each patient in each phase of the cell cycle were analyzed using Hotelling's *T*-test. This multivariate analysis tests the null hypothesis that the proportion of cells in each phase of the cell cycle is the same in MDS and orthopedic control patients. When this test was significant, we further tested whether the proportions of cells in each cell cycle phase were the same in MDS and orthopedic control patients. We used independent *t*-tests with unequal variance for these comparisons. Following Fisher's protected least significant difference approach, we did not adjust these univariate tests for multiple comparisons.

Differences in expression of p-H2A.X and Annexin V between MDS and control subjects were assessed using the Wilcoxon rank sum test.

## 3. Results

### 3.1. Demographic and Diagnostic Data

Patients' (*n* = 19) demographic and clinical lab data (Table 1) are unremarkable for MDS patients. The median age of patients was 58.5 years (range 5–82 years), with a male to female ratio of 1.4 : 1. Mean patient hemoglobin, hematocrit, platelet count, and white blood cell count all differed significantly from control values. MDS patients had intermediate- (refractory cytopenia with multilineage dysplasia, RCMD, *n* = 9; or RCMD with ringed sideroblasts, RCMD-RS, *n* = 1) to high-grade (refractory anemia with excess blasts-1, RAEB-1, *n* = 2; or RAEB-2, *n* = 5) disease (WHO) [9]. Two patients (including the single treatment-related case) had marrow reticulin fibrosis, precluding precise evaluation of dysplasia and blast percentage. Both had pancytopenia and were not low-grade MDS. IPSS scores were Low (*n* = 3), Int-1 (*n* = 6), Int-2 (*n* = 7), or High (*n* = 3) [11]. The control group of patients were demographically similar to MDS patients (median age 61.3, range 45–92 years), with a male to female ratio of 0.7 : 1, showing a slight bias of controls to female patients.

### 3.2. Cell Cycle Analysis

The approach for collection of cell cycle fraction results for each hematopoietic lineage is shown for a representative control patient in Figure 1. 

CD45 versus side scatter plots produce clusters that represent major lineages [15]. We used CD34 positivity to identify immature cells (blue) and CD71 positivity for nucleated erythroid precursors (red). CD45 versus side scatter alone identified lymphocytes (orange) and myeloid precursors (green). DRAQ5 uptake allowed restriction of analysis to nucleated cells and provided cell cycle phase fractions based on DNA content (insets). MPM-2 reactivity was used to identify mitotic cells. As demonstrated in Figure 1, by both the ratio of S and G2 to G0/G1 (insets) and the fraction of mitotic cells (arrows), different lineage sets have different phase fraction profiles, suggesting that they cycle at different rates, following the order erythroid > myeloid > CD34+> lymphoid from most rapidly proliferating to slowest. Mean results and ranges for cell cycle distribution in the control set are summarized in Table 2. 

**Figure 2:**
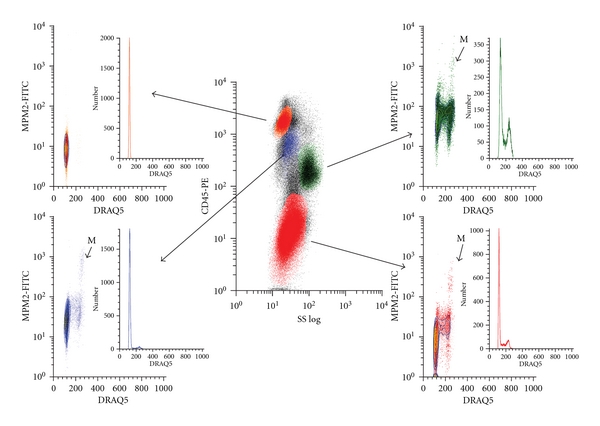
Multiparametric flow cytometry analysis of representative MDS bone marrow. Multiparametric flow cytometry analysis of a representative MDS case using the same analytical scheme and color coding as described in Figure 1. G-M progenitors (green) contain an accumulation of cells in G2 with diminished mitosis (M) compared to G-M progenitors in the control marrow (Figure 1). In this case erythroid precursors (red) and CD34+ stem cells (blue) exhibit relatively normal cell cycle parameters (DNA content, mitotic cells) versus the control sample (Figure 1).

A representative MDS patient is shown in Figure 2. Mean results and ranges for cell cycle distribution in MDS patients are summarized in Table 2. 

The proportion of cells in each cell cycle phase was compared between case and control subjects for each hematopoietic cell type. These proportions were not significantly different for either the erythroid progenitors or CD34+ cells. However, they did differ significantly for myeloid progenitors (*P* = .002). Among MDS patients the average proportion of myeloid cells in G2 was 4.3% versus 1.6% in control patients (*P* = .004; see Table 1). Notably, mitotic events were not significantly elevated in any MDS patient cell group (erythroid, myeloid, or CD34+) compared to controls. 

### 3.3. H2A.X Phosphorylation

**Figure 3:**
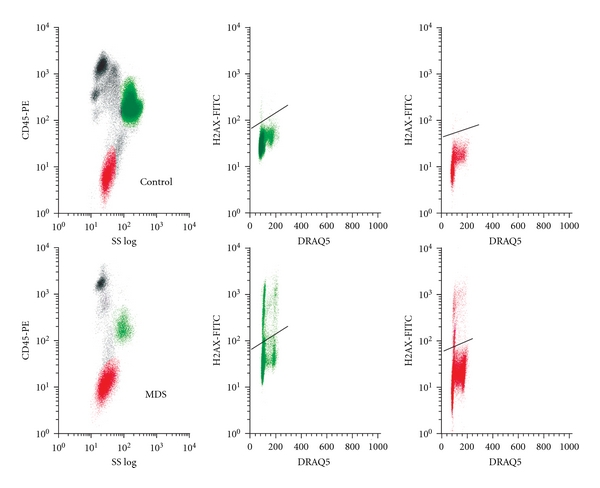
Comparative *γ*H2A.X findings in representative control versus MDS marrow. Multiparametric flow cytometry analysis of *γ*H2A.X density and DRAQ V in G-M and erythroid progenitors in control (top) and MDS (bottom) marrows. Major subsets of cells were identified as described in Figure 1 for control (top left) and MDS (bottom left). G-M progenitor cells from control (top middle) and MDS (bottom middle) and erythroid progenitor cells from control (top right) and MDS (bottom right) were analyzed for DNA and *γ*H2AX. The horizontal bars were used to determine levels of *γ*H2AX density in MDS above the control marrow in both G-M and erythroid progenitors.

Because of our finding of an elevated G2 fraction in myeloid cells in MDS, we evaluated phosphorylation of histone H2A.X at serine 139 (p-H2A.X), a marker of DNA double-strand breaks (DSBs), to explore the possibility of excessive DNA damage in MDS cells. Representative displays of p-H2A.X reactivity versus DNA content in a control and an MDS patient are shown in Figure 3, with p-H2A.X reactivity within each cell subtype summarized in Table 3 [20, 21]. While there is heterogeneity among samples (see ranges, Table 3), we detected a 5-fold increase of p-H2A.X reactivity in the erythroid compartment in MDS versus controls that may indicate an increase but did not rise to statistical significance (*P* = .20). A more marked 14-fold increase in myeloid progenitors versus controls was significant (*P* = .001). CD34+ cells showed no significant difference of MDS samples compared to controls. 

### 3.4. Annexin V

**Figure 4:**
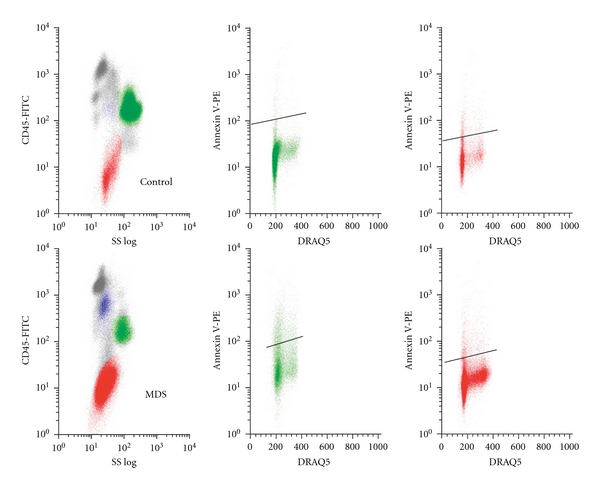
Comparative Annexin V findings in representative control versus MDS marrow. Multiparametric flow cytometry analysis of Annexin V positivity and DRAQ V in G-M and erythroid progenitors in control (top) and MDS (bottom) marrows. Major subsets of cells were identified as described in Figure 1 for control (top left) and MDS (bottom left). G-M progenitor cells from control (top middle) and MDS (bottom middle) and erythroid progenitor cells from control (top right) and MDS (bottom right) were analyzed for DRAQ V and Annexin V. The horizontal bars were used to determine levels of Annexin V positivity in MDS above the control marrow in both G-M and erythroid progenitors.

Since increased apoptosis has been associated with MDS in some reports, [22] we evaluated apoptosis using Annexin V binding in each cell compartment. Representative displays of Annexin V reactivity versus DNA content in a control and an MDS patient are shown in Figure 4, with Annexin V reactivity within each cell subtype summarized in Table 3. There is heterogeneity among hematopoietic cell subsets, but we did not identify any significant increase in the proportion of apoptotic cells in any hematopoietic cell subset in MDS compared to the control population. 

### 3.5. Cell Cycle-Related H2A.X Phosphorylation and Annexin V Reactivity

DNA fragmentation during apoptosis can result in H2A.X phosphorylation, although usually the level of phosphorylation is much greater than that measured for DNA damage [20, 21]. We analyzed the relationship of H2A.X phosphorylation versus Annexin V binding. Assessment of H2A.X phosphorylation versus DNA content, illustrated in representative control and MDS samples in Figure 3, allowed analysis of p-H2A.X reactivity by cell cycle compartment (G0/G1, post-G1) in erythroid and myeloid progenitors using combinations of p-H2A.X reactivity, side scatter, CD45 antigen density, and DNA content. Control erythroid progenitors demonstrate minimal p-H2A.X reactivity, while MDS samples demonstrate p-H2A.X reactivity, predominantly in G0/G1 but also in S and G2. Control myeloid progenitors demonstrate modest p-H2A.X reactivity, predominantly in G0/G1, while MDS samples demonstrate marked reactivity in G0/G1, S, and G2. Similar cell cycle analyses for Annexin V are illustrated in representative control and MDS samples in Figure 4. In contrast to p-H2A.X reactivity, Annexin V demonstrates similar cell cycle distribution patterns in MDS and control samples, with predominant expression in G0/G1. These analyses allowed comparison of the interrelationship of p-H2A.X and Annexin V reactivity in cell cycle fractions. p-H2A.X and Annexin V reactivity correlate significantly only in G0/G1 in myeloid progenitors (*P* = .05); post-G1 myeloid progenitor and both G0/G1 and post-G1 erythroid progenitor p-H2A.X and Annexin V reactivities demonstrate no correlation. These results suggest that at least a major proportion of H2A.X phosphorylation at serine 139 in MDS is unrelated to apoptosis, neither a result of DNA fragmentation during apoptosis nor associated with initiation of apoptosis.

## 4. Discussion

MDS is characterized by life-threatening peripheral cytopenias and a tendency to progress to a subset of AML that is difficult to treat. Improvements in treatment approaches for MDS and this subset of AML may require clarification of the pathogenesis of MDS. A current pathogenetic model characterizes MDS as a combination of hyperproliferation of marrow progenitors with excessive apoptosis, leading to poor production of peripheral blood cells. An alternative model characterizes MDS as a mutator phenotype with DNA damage as the direct cause of both peripheral cytopenias due to impairment of cell cycling of hematopoietic progenitors and leukemic progression. To evaluate these possibilities, we analyzed marrow samples from MDS patients using multiparametric flow cytometry techniques that allowed analysis of the cell cycle, including mitotic activity, in individual hematopoietic precursor compartments, and additional analysis of DNA damage and apoptosis in both individual hematopoietic precursor compartments and cell cycle subsets. Of particular note, neither erythroid, myeloid, nor CD34+ cells in MDS patients exhibited significantly increased mitotic events versus controls, indicating that MDS marrow is not hyperproliferative. These results call into question previous interpretations of DNA content [23–25] and *in vivo* DNA labeling [26–29] studies as indicative of increased proliferation of marrow cells in MDS. As our data show, a finding of increased cells in S or G2 by DNA content without simultaneous analysis of mitotic activity is not definitive evidence of proliferation. We also demonstrate unexpected heterogeneity in cell cycle fractions in different hematopoietic progenitor subsets in both controls and MDS samples, with erythroid progenitors showing a 1.5- to 2-fold increase in S and G2 events versus myeloid progenitors. Thus, failure to compensate for erythroid hyperplasia, a common finding in MDS, in interpreting DNA content and labeling data could result in misinterpretation of results as hyperproliferation, rather than normal proliferation with an expanded erythroid compartment. Other factors may also be contributory to variance of our results versus *in vivo* DNA labeling studies. Incorporation of labeled nucleotides in DNA is not uniquely specific for DNA synthesis (e.g., it may occur with DNA repair, and our findings suggest increased DNA damage in MDS), and *in vivo* DNA labeling studies can include no normal controls, as the agents are mutagenic.

Under normal physiologic control in mammalian cells, S, G2, and M should maintain constant ratios. Our demonstration of a significant increase in the G2 compartment in myeloid precursors in MDS patients versus controls, with no increase in mitotic events, indicates aberrant cell cycle progression with delayed G2 transit, not proliferation. Mean G2 cells were also increased above controls in erythroid precursors, but the difference was not significant. A possible explanation for delayed G2 transit is DNA damage. To evaluate this possibility, we analyzed MDS samples for histone H2A.X phosphorylation at serine 139 (p-H2A.X), this phosphorylation being mediated by ATM in response to double-strand DNA breaks [20, 21, 30, 31]. We observed a highly significant 14-fold elevation of p-H2A.X in myeloid precursors in MDS samples and a 5-fold (not significant) elevation in erythroid precursors. These findings suggest that hematopoietic precursors in MDS have high levels of DNA damage, a possible explanation for both the delayed cell cycle transit that our data suggest and the preleukemic phenotype of MDS. Horibe et al. have recently reported similar results, using immunohistochemistry to demonstrate activation of ATM and phosphorylation of its substrate H2A.X in marrow samples from MDS patients, with little activity in control marrows. In addition, their observation of activation of the checkpoint genes Chk2 and p53 in MDS is consistent with the delayed G2 transit that our data suggest [32]. 

Using Annexin V reactivity we were unable to demonstrate the increased apoptosis reported in MDS marrow by others (reviewed in [33]). An explanation for this disparity is uncertain. We did support marrow cell viability *ex vivo* with RPMI, coupled with rapid processing and analysis. We found no correlation of p-H2A.X versus Annexin V in most settings. We did note this correlation in G0/G1 in myeloid progenitors. DNA degradation during late apoptosis may result in marked phosphorylation of H2A.X (which we did not detect) [20], while DNA damage (with associated H2A.X phosphorylation) can trigger apoptosis. It is possible that the correlation we observed in this limited setting is due to the latter. 

Our finding of normal mitotic rates in MDS samples is consistent with our Annexin V results, suggesting that our results reflect more accurately the *in vivo* physiologic state in MDS. It would be kinetically impossible to maintain the typical hypercellular marrow in MDS with increased apoptosis but no increase in mitotic activity. It should be noted that assays of apoptosis are performed *ex vivo*. Production of uric acid is an *in vivo* surrogate for cell death; the routine absence of hyperuricemia in MDS is inconsistent with increased *in vivo* apoptosis. Our finding of no apoptotic response in cells in G2 despite evidence of increased DNA damage suggests an alternative interpretation of data in MDS, namely that in MDS there may be a failure* in vivo* to trigger effective apoptosis in response to DNA damage. If so, cells with unrepaired DNA damage could accumulate in marrow, resulting in hypercellularity. With the additional stress of aspiration, these cells would proceed to apoptosis, resulting in a false impression of excessive *in vivo* apoptosis.

Multiparametric flow cytometry was crucial for performance of these studies, allowing simultaneous quantitative analysis of cell cycle and other parameters in electronically segregated subpopulations of marrow cells. Use of MPM-2, with direct quantitation of mitoses, gave a more complete assessment of the cell cycle than DNA content alone [12–14]. Use of DRAQ5 for DNA content analysis allowed simultaneous analysis with other parameters in segregated hematopoietic subpopulations, which demonstrated unexpected heterogeneity of virtually all parameters tested among different hematopoietic subpopulations. Our results cast doubt on the interpretability of whole marrow analyses of these parameters in previously reported studies and allowed demonstration of abnormal cell cycle progression in one hematopoietic subset. We demonstrated differences in results for some parameters (p-H2A.X, Annexin V) in different cell cycle fractions and that these differences also varied in differing hematopoietic progenitor subpopulations. This complexity would be difficult to demonstrate with any other available analytical technology.

## 5. Conclusions

We demonstrate that MDS is not hyperproliferative, that MDS may have an aberrant delay in the cell cycle, that MDS has evidence of unrepaired DNA damage, and that MDS may have a diminished *in vivo* apoptotic response to that damage. These observations are in contradistinction to a widely held model of MDS as a hyperproliferative process with excessive* in vivo* programmed cell death. We hypothesize that DNA damage with aberrant cell cycling may contribute directly to both the marrow failure of MDS and its preleukemic phenotype.

## Figures and Tables

**Table 1:** MDS patient demographic and diagnostic data.

	Age	Sex	Hct	Plts	ANC	Blast %		Cytogenetics	Diagnosis	IPSS
						PB	MRW			
1	58	M	38	66	1.7	0	0	t(1;7)	Hypocellular RCMD	Int-2
2	72	M	24	91	5.5	0	4	(−5, −7)	RCMD	Int-2
3	75	F	25	13	4.3	0	0.5	1 abnormal cell	RCMD-RS	Int-1
4	59	M	32	134	0.8	0	13	(20q-, −5, +14)	RAEB-2	High

5	62	M	28	35	0.9	0	<5	(−7)	2° MDS, retic fibr	Int-2
6	5	F	24	49	0.9	0	<0.5	46XX	RCMD	Int-1
7	59	M	26	12	1.8	0	<0.5	46XY	RCMD	Int-1
8	82	F	24	27	1.1	0	<5	no metaphases	MDS with retic fibr	Int-1
9	65	F	38	97	2.1	0	1.5	(−5)	RCMD	Int-1
10	52	F	21	366	3.5	0	3.5	46XX	RCMD	Low

11	63	M	30	20	0.8	4	11	(−5, −7), other	RAEB-2	High
12	65	F	26	23	0.7	0	6	46XX	RAEB-1	Int-1
13	67	F	24	13	0.03	0	8	(−5, 20q-)	RAEB-1	Int-2
14	58	M	26	66	0.1	0	12	(20q-)	RAEB-2	Int-2
15	64	M	27	51	0.2	0	11	t(3; 21)	RAEB-2	High

16	38	F	35	208	0.7	0	0.5	46XX	RCMD	Low
17	58	M	27	15	0.05	0	11	(20q-)	RAEB-2	Int-2
18	20	M	37	23	0.2	0	<0.5	(−7) (familial)	RCMD	Int-2
19	81	M	30	178	3.5	0	1.5	46XY	RCMD	Low

Hct: % hematocrit; Plts: platelets (thousands/microliter); ANC: absolute neutrophil count (thousands/microliter); PB: peripheral blood; MRW: marrow; retic fibr: reticulin fibrosis; RCMD: refractory cytopenia with multilineage dysplasia; RAEB: refractory anemia with excess blasts; IPSS: International Prognostic Scoring System for MDS; Int: intermediate.

**Table 2:** Cell cycle analysis of marrow samples.

	S (%)		G2 (%)		M (%)	
	Controls	MDS	Controls	MDS	Controls	MDS
nRBC	27.9 (16.2–41.9)	31.2 (12.8–56.4)	4.7 (2.1–8.2)	6.3 (1.1–17.0)	1.1 (0.5–1.6)	1.0 (0.01–3.6)
Myeloid	16.0 (6.7–28.1)	17.16 (7.8–32.1)	1.6 (0.0–3.3)	4.3 (0.2–11.5)	0.32 (0.2–0.6)	0.45 (0.0–1.1)
CD34+	13.5* (7.4–22.9)	10.9* (2.0–16.0)	*	*	0.6 (0.2–1.1)	0.8 (0.1–2.3)

Controls (*n* = 20), MDS (*n* = 19).

Cell populations discriminated by CD45, SS, CD71, and CD34.

DNA content assessed using DRAQ5; mitoses assessed using MPM-2.

Results expressed as mean (%) positive (range).

*Because of infrequent stem cell events, S and G2 are combined as post-G1 for stem cells.

**Table 3:** p-H2A.X and Annexin V reactivity in marrow cells.

	p-H2A.X (%)		Annexin V (%)	
	Controls (*n* = 20)	MDS (*n* = 17)	Controls (*n* = 20)	MDS (*n* = 17)
nRBC	1.5 (0.4–3.7)	8.0 (0.1–49.9)	19.4 (3.1–54.6)	12.3 (2.2–55.3)
Myeloid	1.1 (0.1–4.6)	15.9 (0.2–51.0)	16.4 (4.0–58.0)	24.2 (1.2–89.7)
CD34+	1.7 (0.2–4.9)	1.7 (0.0–5.1)	11.6 (3.8–26.5)	11.9 (1.9–61.5)

Cell populations discriminated by CD45, SS, CD71, and CD34.

Results expressed as mean (%) positive (range).
